# Germline stem cells in human

**DOI:** 10.1038/s41392-022-01197-3

**Published:** 2022-10-02

**Authors:** Hanhua Cheng, Dantong Shang, Rongjia Zhou

**Affiliations:** Hubei Key Laboratory of Cell Homeostasis, College of Life Sciences, Renmin Hospital of Wuhan University, Wuhan University, 430072 Wuhan, China

**Keywords:** Cell biology, Molecular medicine, Pluripotent stem cells

## Abstract

The germline cells are essential for the propagation of human beings, thus essential for the survival of mankind. The germline stem cells, as a unique cell type, generate various states of germ stem cells and then differentiate into specialized cells, spermatozoa and ova, for producing offspring, while self-renew to generate more stem cells. Abnormal development of germline stem cells often causes severe diseases in humans, including infertility and cancer. Primordial germ cells (PGCs) first emerge during early embryonic development, migrate into the gentile ridge, and then join in the formation of gonads. In males, they differentiate into spermatogonial stem cells, which give rise to spermatozoa via meiosis from the onset of puberty, while in females, the female germline stem cells (FGSCs) retain stemness in the ovary and initiate meiosis to generate oocytes. Primordial germ cell-like cells (PGCLCs) can be induced in vitro from embryonic stem cells or induced pluripotent stem cells. In this review, we focus on current advances in these embryonic and adult germline stem cells, and the induced PGCLCs in humans, provide an overview of molecular mechanisms underlying the development and differentiation of the germline stem cells and outline their physiological functions, pathological implications, and clinical applications.

## Introduction

In mammals, an organism consists mainly of two cell types, somatic cells, and germ cells. Based on the concept of the germline proposed by early biologist August Weismann,^1^ the somatic cells die along with the individual, in contrast, the germ cells can pass both genetic and epigenetic information from one generation to the next. The germline is a lineage of cells in an organism from which both oocytes and sperm cells arise, which is thus essential for the propagation of species. As such, abnormal development of the germline cells will lead to severe diseases in humans, including infertility and cancer. For example, the incidence rate of ovarian cancer is 11.5 per 100,000 women during 2010–2014,^2^ there are 22,530 new cases in 2019,^3^ and an estimated 19,880 people will be diagnosed with ovarian cancer in the United State.^4^ Ovarian cancers are also among incidences of top 10 cancers for females, with 50,000 cases in China.^5^ In females, an increasing prevalence of disease type is ovarian dysfunction, which includes altered frequency, and duration of the menstrual cycle,^6^ with or without premature ovarian failure or polycystic ovary syndrome. Testicular germ cell cancers account for ~1% of all solid cancers in Caucasian males, in particular, 60% are diagnosed in adolescents and young adults.^7–9^ In addition, infertility is common and affects around 8–17% of reproductive-aged couples worldwide.^6,10,11^ Thus, the development and regulation of germline cells play a fundamental role in survival, health, and disease in humans.

During early embryonic development, first emerged germline cells are called primordial germ cells (PGCs).^12^ The PGCs are the founder cells of the germline, to some extent, also the source of germline totipotency, ensuring the creation of new organisms.^13^ In humans, when, where, and how first PGCs are specified within an early embryo remain a central challenge. There are probably two sets of constraints for this: One is ethical and technical limitations in obtaining and manipulating human (h) PGCs (hPGCs) from early embryos at the peri-implantation stage, and the other is differences in PGC development among species for comparative studies. While animal models have provided instructive knowledge for hPGCs, cell and molecular mechanisms underlying PGCs specification observed in model animals are generally incapable of recapitulating essential points of those in humans.

In humans, early investigations by light microscopy found that hPGCs have a large size with a large nucleus and prominent nucleolus in the yolk-sac endoderm of embryonic day 24 (E24), which migrated into the developing genital ridges at E28.^14^ Witschi suggested that the hPGCs migrated within the embryo by active movements,^14^ which was confirmed by a time-lapse analysis of living mouse (m) PGCs (mPGCs) migration 50 years later.^15^ However, passive movement associated with morphogenesis has also been observed.^16^ Fine morphology, migration, and origin of hPGCs were then observed by transmission electron microscopy, which was characterized by the presence of abundant glycogen particles and lipid droplets in the cytoplasm.^17^ This observation has metabolic implications for hPGCs, as the term glycolysis is often used to describe stemness.^18^ Alkaline phosphatase activity was also observed on the plasma membrane of the hPGCs, indicating a characteristic marker of hPGCs.^17,19^ To overcome technical limitations in manipulating early hPGCs in vivo, approaches of in vitro induction of hPGCs have been established from embryonic stem cells and induced pluripotent stem cells.^20–22^ These induced hPGCs are called hPGC-Like Cells (hPGCLCs), which are a bona fide in vitro counterpart of hPGCs.^23^ Lineage trajectory and mechanistic insights of hPGCs specification using the hPGCLCs induction system have been further clarified by means of single-cell transcriptomics and cell lineage tracing.^24^ Recently, the hPGCLCs have also been used for in vitro gametogenesis,^23^ which has important implications in reproductive medicine.

As precursors of the gametes, hPGCs continue to divide mitotically when arriving at genital ridges. In the following processes of gonad development, hPGCs will go through a distinct process of development depending on their sex chromosome composition (XX/XY) in embryos. In female embryos (XX), some hPGCs enter into the meiotic division phase and subsequently differentiate into oocytes in the ovary, thus ending their stem cell potential, while the others keep stemness and become FGSCs.^25^ In contrast to those in the female, male hPGCs (XY) enter the seminiferous cords, become gonocytes, and are arrested in G0/G1 phase of cell cycles until birth.^26–28^ In neonatal testis, the gonocytes resume to divide and differentiate into spermatogonial stem cells (SSCs), which then give rise to spermatozoa via meiosis from the onset of puberty. Manipulation and transplantation of the SSCs provide a powerful system to study stem cell biology, preserve individual genomes, modify germ lines and treat male infertility.^27,29,30^ For example, SSCs transplantation can recover male fertility when the SSCs of patients are damaged upon irradiation or chemotherapy in cancer treatment.

In mammals, it has long been believed that the total number of ovarian follicles is determined during the perinatal period, and production of ovarian oocytes is thought to stop in adult female.^31–33^ However, accumulating evidence shows that there are female germline stem cells (FGSCs) in ovaries in mice and humans, which are able to undergo postnatal neo-oogenesis.^25,34–36^ The newly found FGSCs provide an alternative way to investigate the development of germline stem cells by oogenesis, not just by spermatogenesis. More importantly, FGSCs have important clinical implications, for example, in the expansion of the follicle reserve for fertility preservation and treatment of infertility and premature ovarian failure.

**Fig. 1:**
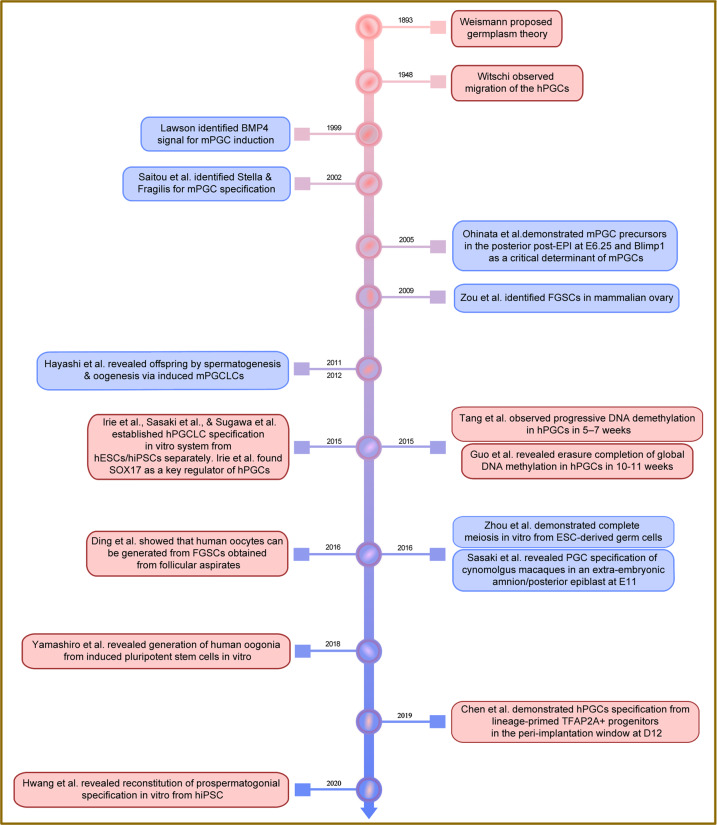
History and main events of the studies in human germline stem cells. A glance of the discovery and advances starts from 1893 and the most of advances in human germline stem cells have been made since 2015

Given the above historical and developmental overview (Fig. 1), the germline stem cell (GSC) is a unique cell type that produces more stem cells via self-renewal or different states/subtypes of stem cells during germline development, and finally differentiate into specialized cells, spermatozoa and ova, for producing offspring. In mammals, the GSCs mainly include (1) primordial germ cells (PGCs) from embryos, being embryonic pluripotent stem cells, (2) induced PGC-like cells (PGCLCs) from embryonic stem cells (ESCs) or induced pluripotent stem cells (iPSCs), (3) spermatogonial stem cells (SSCs), and (4) female germline stem cells (FGSCs). Both SSCs and FGSCs belong to adult pluripotent stem cells (ASCs). Here, we summarize these germline stem cells in humans, provide an overview of molecular mechanisms underlying GSC development and disease, and outline their physiological functions, pathological implications, and potential clinical applications.

## Precursors for the gametes: human primordial germ cells

### Origin and specification of hPGCs

#### Development of human early embryos

**Fig. 2:**
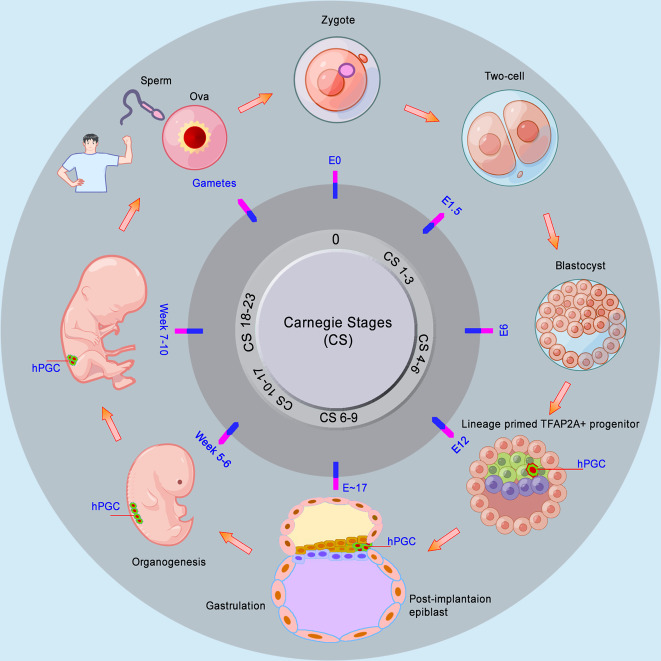
Development of human embryos and the timing of hPGC specification. Human early embryos must obtain essential nourishment from mother by implantation into uterus, which occurs at the end of blastocyst stage. The Carnegie stages (CS) can be corresponding to days after fertilization or embryonic day (E). hPGCs are indicated in green (cytoplasm) and red (nucleus). The main parts of the figure were drawn by Figdraw

Primordial germ cells are early embryonic pluripotent stem cells. Embryonic development begins after fertilization. Eggs support the following embryonic development until the new organism can feed on most animals, including zebrafish, frogs, and chickens. In contrast, in mammals, early embryos must obtain essential nourishment support from the mother by implantation into the uterus. In humans, implantation occurs at the end of the blastocyst stage during embryonic day E7–E8.^37,38^ The inner cell mass splits into the hypoblast, which forms the yolk sac, and the epiblast, which generates the embryo properly. The post-implanted embryo in humans is flat with a bilaminar germ disc, consisting of the epiblast and hypoblast, in addition to the trophoblast, whereas it is cylindrical in mice.^39,40^ The following formation of the primitive streak begins in the posterior part of the embryonic epiblast at E14, which means the start of gastrulation, and the cells gradually lose their pluripotency. Then the epiblast cells move through the primitive streak to give rise to mesoderm and endoderm. At the end of gastrulation, the other epiblast cells become ectoderm.^38,39^ The formation of the three germ layers starts the subsequent organogenesis of embryos. The timing of human embryo development is often referred to as Carnegie stages (CS), based on the appearance of morphological structures rather than time.^41^ Nevertheless, the CS can loosely be corresponding to days after fertilization or embryonic day.^37,38,42–44^ Based on this, the timing of hPGC development is determined (Fig. 2).

#### Origin of hPGCs

Primordial germ cells are the first population of germ cells established during early embryo development in animals,^12^ yet the origin of PGCs differs among animals. Single-cell transcriptome shows that the germline of *Xenopus* is evolutionarily closer to that of zebrafish than to humans and mice.^45^ In some model organisms, including *Xenopus*,^46–58^ zebrafish,^59–69^ and chicken,^70–82^ their PGCs are specified via the maternally inherited germ plasm, including vasa and nanos, during the first several cleavages. In contrast, in mammals, the PGCs are specified by induction during early embryo development. In mice, the PGCs were observed at the base of the incipient allantois in the extraembryonic mesoderm (ExM) at E7.25.^12,83,84^ Lineage tracing shows that the mPGC precursors with Blimp1+ were detected in the posterior post-epiblast (EPI) at E6.25, indicating the origin of the mPGCs from the posterior post-EPI in mice.^85,86^ In cynomolgus monkeys, cyPGCs are specified in the early amnion at E11 prior to gastrulation.^87^ Nevertheless, it has been a long journey to determine the origin of hPGCs in human embryos. Early observations by histology and microscopy of human embryos showed the hPGCs identifiable as early as E24.^14,17,19^ Recently, the origin of hPGCs was accurately determined by single-cell RNA sequencing and lineage trajectory mapping of the hPGCLC. The study shows that hPGCs were specified beginning at E12 from lineage-primed TFAP2A+ progenitors, which share characteristics with pre- and postimplantation epiblasts^24^ (Fig. 2). In addition, rabbit PGCs show a similar developmental pattern and mechanism with hPGCs, including bilaminar-disc embryos, PGC origin of epiblasts, and SOX17 as a key regulator of PGCs,^88^ suggesting a valuable model for studies of human PGC development.

### Regulation of hPGC specification

#### Specification genes of hPGCs

Knowledge about how PGCs are induced comes largely from studies in model mammals, including nonhuman primates,^86,87,89–95^ mice,^12,83,85,96–108^ and pigs.^109–118^ Studies in the other group of model organisms, including chicken,^119–126^ zebrafish,^63,64,66,67,127^ medaka,^128–136^ frogs,^55–57,137^ *Drosophila,*^138–146^ and *Caenorhabditis elegans,*^147–156^ provide abundant information about PGC specification by preformation or maternally inherited determinants. The PGCs in these two groups of organisms show a similarity that is the presence of some kind of aggregate of electron-dense, basophilic bodies containing the proteins and RNAs, (e.g., VASA, NANOS, and MAGO) in their cytoplasm.^70^ Nevertheless, even between mice and humans, accumulated evidence shows that there are differences in cell and molecular mechanisms underlying PGC specification, especially, in expressed genes and signaling pathways.^24,86,157^ For example, SOX17 is critical for hPGC specification, but not for mPGC induction.^20^ SOX2 is expressed in mPGCs, but not in hPGCs.^158^ In humans, SOX2 exerts its roles mainly in adult tissues and cancers through regulating self-renewal and stemness of cancer stem cells.^159^ In recent years, studies in nonhuman primates provide some information for hPGC specification.^87,89,91,95,160^

In PGCs of humans and nonhuman primates, a core group of primate-specific PGC markers is expressed, including SOX17, BLIMP1, TFAP2C, NANOG, and POU5F1, but the absence of SOX2,^87,90,95,160,161^ suggesting that these factors are conserved and are important for PGC specification in primates. Nevertheless, it is perhaps different from the origin of hPGCs, that PGCs in cynomolgus monkey might emerge in amnion.^87,95^ In general, hPGCs/hPGCLCs express a range of types of key genes, including (1) pluripotency markers: NANOG, POU5F1, ALPL, KLF4, LIN28, KIT, NANOS3, SSEA-1, SSEA-4, DPPA3 (also called as STELLA), and ZFP42 (also known as REX1),^17,20,21,161–167^ (2) cell-surface makers: CD38, EPCAM, ITGA6 (INTEGRIN alpha 6), ITGB3, FGFR3, KIT, and ALPL,^20,21,167,168^ (3) germline markers: SOX17, BLIMP1 (also known as PRDM1), TFAP2C, PRDM14, DDX4 (also known as VASA), DAZL, and TCL1A,^20,21,167,169–172^ (4) amnion-related genes: CDX2 and GATA3,^24^ (5) mesoderm markers: EOMES, NODAL, SP5, and T,^20,21^ (6) transcription factors: SOX17, BLIMP1, SOX15, GATA4, PRDM14, SALL4, and UTF1,^20,21,172–175^ and (7) epigenetic regulation factors: DNA demethylation dioxygenases (TET1, TET2, and TET3), protein arginine methyltransferase 5(PRMT5), and DND microRNA-mediated repression inhibitor 1(DND1).^20,167,176,177^ The types and expression patterns of these marker genes reflect corresponding states of hPGC development and cell identity. For example, expression levels of pluripotency genes gradually decrease in hPGCs from embryos of 4–19 weeks,^167^ indicating a slow loss of pluripotency. Thus, not all these types of genes are expressed in a certain state of hPGC at a certain developmental time. Of course, there are other factors important for hPGC development, that remain to be identified. For example, TRIM71, an E3 ubiquitin ligase, is associated with the proliferation of hPGC-like TCam-2 cells.^178^ NOD-like receptor Nlrp14 knockout inhibits SSC differentiation in mice.^179^

#### Signaling pathways and regulations for hPGC specification

##### BMP-SMAD signaling

The bone morphogenetic proteins (BMP) are members of the transforming growth factor-beta superfamily and play important roles in embryo development.^180^ Further studies in knockout mice reveal that BMP proteins are required for PGC induction.^96,107^ Some upstream factors that regulate BMP expression play important roles in PGC formation, for example, LncBMP4, a long noncoding RNA that targets BMP4, has similar functions as BMP4.^119^ BMP proteins originate from the extraembryonic tissues but exert their roles through their receptors (BMPRs) on the membrane of epiblast cells. Upon binding to BMPRs that phosphorylate intracellular signaling molecules SMAD1/SMAD5 in the cytoplasm, the activated SMAD1/SMAD5 dimerize with SMAD4, translocate into the nucleus, and regulate the key transcriptional regulators of PGCs.^98,181^ In mice, BMP4 induces PGCs with an expression of both BLIMP1 and PRDM14 in the epiblast, which can be induced to generate functional sperm cells in vivo by gonad reconstruction and seminiferous tubule injection.^13^ *Bmp4* homozygous KO embryos lack PGC development, demonstrating a key role of BMP4 in PGC induction in vivo.^96^ The authors also showed that the response of epiblast cells to BMP is dose-dependent during PGC induction. Further analysis of the roles of intracellular signaling molecules in PGC induction indicates that SMAD1 signaling is critical for the initial commitment of PGCs, as evidenced by the fact that the knockout of *Smad1* led to the complete absence of PGCs in mice.^98^ In cultured epiblast cells, BMP4 is also sufficient to induce PGCs in a dose-responsive manner.^13^ In a culture of hPGCs from fetal gonads at 8–11 weeks, the addition of BMP4 increases the number of hPGCs in a dose-responsive manner, whereas the addition of an antagonist of the BMP4 pathway decreases PGC proliferation.^182^ WNT3 is expressed in the epiblast at around E5.5 and this ensures its responsiveness to BMP4 signalling.^13,183^ In addition, activin A induces the expression of OCT4, NANOG, NODAL, WNT3, bFGF, and FGF8, and suppresses the BMP signaling,^184^ but it shows high competence to differentiate to hPGCLCs when transiently converted to the 4i-state prior to differentiation in culture,^185^ thus induces an increased differentiation potential of germ cells.^186^ These studies clearly suggest that BMP-SMAD signaling is key and indispensable for PGC specification in mammals. Thus, in the following studies of hPGCLCs induction in vitro, BMP4 is widely used as an essential factor.^20,21^ Upstream regulation of BMP4 will be another layer for hPGC specification. In nonhuman primates, transcription factor ISL1 acts upstream of BMP4 and plays an indispensable for amnion formation.^187^ Given amnion as a signaling center during mesoderm formation, it is possible that ISL1 might function in hPGC specification, which needs to be explored further.

##### SOX17- BLIMP1

SOX17, a member of the SOX (SRY-related HMG-box) family of transcription factors, is originally identified as a transcription factor for spermatogenesis.^188,189^ Later studies reveal an important role of SOX17 in endoderm development of the post-implanted embryos in mice, as knockout embryos are deficient in gut endoderm.^190^ Induction in vitro of hPGCLCs reveals that SOX17 is a key regulator of hPGC fate, loss of SOX17 impairs hPGC specification, and BLIMP1 works downstream of SOX17, which then represses endodermal and somatic genes.^20^ This pathway works only in hPGCs, but it is not necessary for mPGC fate. The transcription factor EOMES (T-box gene Eomesodermin) functions upstream of SOX17 for hPGCLC specification, which is upregulated in incipient mesoderm-like cells (iMeLCs) and activates SOX17 in response to WNT signaling.^191^ EOMES knockout impairs hPGCLC differentiation from human embryonic stem cells (hESCs).^192^ These data suggest an essential role of EOMES for hPGCLC specification. In mice, loss-of-function reveals that *Eomes* mutants arrest at implantation, suggesting a critical role of EOMES in the specification of the definitive endoderm lineage.^193^ The mesodermal protein T (TBXT) is a downstream effector of WNT3 signaling and essential for mPGC specification in mice,^194^ but it is dispensable for hPGCs.^191^ Further studies revealed that SOX17, TFAP2C, and BLIMP1 are not sufficient to generate hPGCLCs, in contrast, transcription factors GATA3/GATA2 as key BMP effectors, combined with SOX17 and TFAP2C, drive the hPGCLC program.^195^ Nonetheless, the precise molecular mechanisms involved in the transcription factors during hPGC specification in vivo remain to be explored further.

BLIMP1, also known as PRDM1, encodes a zinc finger transcriptional repressor required for anterior endomesodermal cell fate and head induction^196^ and can bind directly to repress somatic cell proliferation genes.^174^ In mice, BLIMP1 is expressed in the most proximal layer of the epiblast at E6.25, BLIMP1-positive cells are lineage-restricted to mPGCs, and in Blimp1 mutants, formation and immigration of mPGC are impaired.^85^ But, BLIMP1 is dispensable for the derivation and maintenance of ESCs and postimplantation epiblast stem cells.^197^ In humans, BLIMP1 expression is detected in human fetal gonocytes in 12th week.^176^ Accumulated evidence shows that BLIMP1 is essential for hPGCLC specification,^20–22,171,191^ and this function is also conserved in mPGCs.^85,198^ Mechanistically, BLIMP1 acts downstream of SOX17 to suppress neuron differentiation and both endodermal and mesodermal genes and initiate the transcriptional network of human germ cells, including NANOS3.^20,21,195^ In the knockout cells of BLIMP1 or SOX17, the gene expression network of hPGC specification is also abrogated, including NANOS3.^20^ Thus, the SOX17-BLIMP1 axis initiates hPGC program from competent cells upon induction by BMP signaling.^20^ As a complex and programmed developmental process, hPGC development needs many other genes and a coordinated network. For example, another two transcription factors TFAP2C and PRDM14 play indispensable roles in hPGC specification.

##### TFAP2C

TFAP2C (also known as AP2-GAMMA) is a sequence-specific DNA-binding transcription factor involved in the activation of several developmental genes. In hESCs, TFAP2C binds to a naive-specific POU5F1 (OCT4) enhancer to maintain pluripotency and repress neuroectodermal differentiation during the transition from primed to naive in preimplantation embryos.^199^ In humans, TFAP2C functions upstream of SOX17 for germline specification, through binding to SOX17 promoter.^24^ On both sides of the binding site, there is also the coordinately enriched H3K27ac in hPGCLCs, and this kind of epigenetic regulation of TFAP2C might enable SOX17 expression at the point of hPGCLC specification.^24^ Through lineage tracking and mapping of the human germline trajectory, Chen and collaborators demonstrated that the TFAP2A-expressing progenitors exhibited the potential for both hPGC specification and amnion/gastrulation development at around day 11, and loss of TFAP2C led to exiting of the germline pathway, but toward differentiation of primitive streak or amnion-like somatic cells.^24^ One of the mechanisms of the TFAP2C-regulated hPGC formation is through the opening of enhancers proximal to pluripotency factor OCT4.^199,200^ Thus, these data suggest that TFAP2C plays an essential role in hPGC specification by directly regulating SOX17 expression at the critical point of hPGC specification. Of particular note is that BMP signaling also activates TFAP2C in a SOX17-independent manner, and both SOX17 and TFAP2C act upstream of BLIMP1 in human germ cell specification.^191^ In mice, the Tfap2c knockout impaired mPGCLC generation from ESCs,^201^ suggesting a similar role of TFAP2C in the maintenance of the PGC specification. However, there is a difference in regulatory mechanisms of TFAP2C in PGC specification between hPGCs and mPGCs. For example, in mice, TFAP2C is a direct target of BLIMP1, which cooperates with PRDM14 to induce PGC gene expression.^174,191^ Yet, TFAP2C regulates other cellular processes, including cell cycle (CDKN1A/P21 and CDK6), in addition to germline development (NANOS3 and c-KIT) in mice.^201^ Together, TFAP2C plays a critical role in PGC specification, but through distinct regulation modes between mice and humans.

##### PRDM14

As a member of the PRDI-BF1 and RIZ homology domain containing (PRDM) family of transcriptional regulators, PRDM14 is expressed in preimplantation embryos and PGCs in mice and humans.^20,22,202,203^ In hPGC-competent pluripotent cells, PRDM14 is highly expressed.^172^ Accumulating evidence shows that PRDM14 plays important roles in the maintenance and induction of pluripotency of stem cells and PGC development in a range of species, including humans,^22,172,204,205^ mice,^174,202,203,206–212^ rats,^213^ and chicken.^214^ In mice, PRDM14 has critical roles for mPGC specification by upregulation of germline-specific genes, suppression of somatic genes, regulation of global epigenetic reprogramming, for example, maintenance of global DNA hypomethylation, histone modifications, and X-chromosomal reprogramming.^174,202,206,212,215–218^ In human ES cells, knockdown of PRDM14 induced expression of early differentiation marker genes and suppressed expression of stem cell markers, whereas overexpression of PRDM14 showed a remarkable suppression of the expression of differentiation marker genes, suggesting a role of PRDM14 in the maintenance of pluripotency in human ES cells by suppressing of expression of differentiation genes.^204^ A genome-wide RNAi screen shows that PRDM14 binds to the proximal enhancer of pluripotency gene POU5F1/OCT4 to regulate its expression in human ES cells, and functional analysis reveals that PRDM14 is also required for reprogramming of fibroblasts to iPSCs.^219^ Nevertheless, the hPGCLCs induced from hPSCs show a minimal PRDM14 expression, which is different from that observed in mPGCs, suggesting that human PGCs could not require PRDM14, or, alternatively, that low levels of PRDM14 expression is enough for hPGC development.^22^ Indeed, inducible loss of PRDM14 affects the efficiency of specification and leads to downregulation of hPGC marker genes, including UTF1 and NANOG, and re-expression of PRDM14 rescues hPGCLC differentiation,^172^ suggesting a critical role of PRDM14 in hPGC fate. Notably, PRDM14 regulates hPGC development probably through coordination with both TFAP2C and BLIMP1, as it shares a subset of transcription targets with TFAP2C and BLIMP1,^172^ although the exact position of PRDM14 in the regulatory network of hPGC specification remains unknown.

##### Regulation network of hPGC specification

In response to BMP4, hPGCLCs can be induced from hESCs.^20^ Transcription factors GATA3 and GATA2 are BMP effectors and promote the hPGCLC specification, together with SOX17 and TFAP2C. BMP signaling could also activate SOX17 and TFAP2C expression, probably independent from GATA3/2.^195^ SOX17 is a critical regulator of hPGCLCs.^20^ BLIMP1 is activated by SOX17, suggesting that SOX17 acts upstream of BLIMP1.^20,191,195^ In addition, BLIMP1 promotes germline transcription and represses the neuronal differentiation program.^21^

**Fig. 3:**
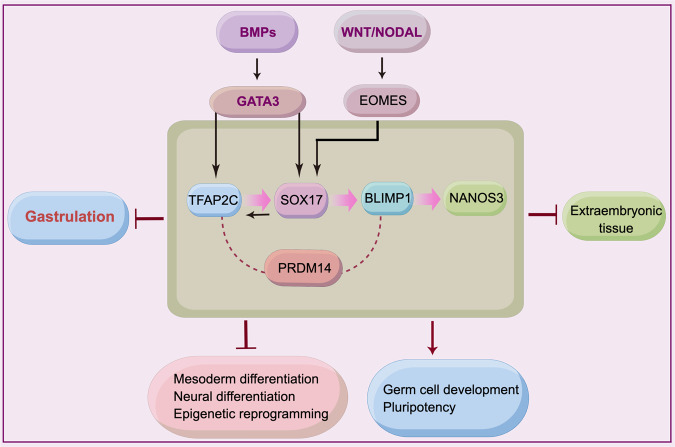
Regulation network of hPGC specification. BMP- and WNT signaling promote the hPGCLC specification via regulating TFAP2C and SOX17. SOX17 is a critical regulator for hPGC specification and works upstream of BLIMP1. TFAP2C activates SOX17 expression. Final effects of hPGC specification promote germline development and pluripotency, while suppress somatic programs. The figure was drawn by Figdraw. Arrows and blunt-ended arrows depict positive and negative regulation, respectively. Dashed lines indicate synergetic role

SOX17 and BLIMP1 together are necessary and sufficient for inducing PGCs and initiating the germline-specific epigenetic program.^109^ TFAP2C activates SOX17 expression through binding to SOX17 promoter, indicating that TFAP2C functions upstream of SOX17 for germline specification.^24^ A recent report shows that SOX17 and TFAP2C activate the expression of PRDM1, POU5F1, and NANOG.^220^ PRDM14 cooperates with TFAP2C and BLIMP1 to induce hPGCLC formation, yet repress WNT signaling and somatic markers.^172^ Nodal signaling is also required for PGCLC specification.^221^ In addition, SOX17, TFAP2C, and PRDM14 upregulate expression of themselves, respectively.^172,195^ The synergetic effects of these factors, on the one hand, induce PGC fate and ultimately, on the other hand, suppress the somatic program (Fig. 3).

##### Epigenetic reprogramming of hPGCs

Epigenetic reprogramming is another layer of regulation in hPGC development. During hPGCs development, shortly after specification, throughout the migration, and towards gonad colonization, epigenetic reprogramming takes place in hPGCs. At genomic DNA levels, globe genomic DNA demethylation is one of the major epigenetic events during hPGCs development, which occurs at week 7.^167^ The inactivated X chromosome is reactivated in female hPGCs of 5.5–9 weeks,^167,171^ which is similar to that in mPGCs.^222^ The lowest level of hypomethylation occurs at week 10 (female) and week 11 (male), respectively.^167^ The low levels of methylation are maintained till week 19, but global re-methylation already starts in female PGCs at week 11 and male PGCs at week 19, respectively.^167^ Consistent with this in hPGCs, DNA demethylation dioxygenase TET1 is highly expressed in hPGCs from week 4 to 11, TET2 and TET3 are also mildly expressed, while the 5hmC level is very low in 7–11 weeks.^167^ A similar pattern of globe DNA demethylation is also detected in hPGCLCs.^20^ As loss of BLIMP1 affects the initiation of DNA demethylation, SOX17 and BLIMP1 pathway is proposed to drive extensive DNA demethylation and chromatin reorganization in hPGC specification.^171^ Nevertheless, global changes in gene expression might not correlate with global changes in DNA methylation in developing prenatal germline cells.^223^ In addition, chromatin modifications are involved in hPGC specification. The hPGCs at week 4 show a remarkable enrichment of H3K27me3, then a declined trend, and hPGCs of 7–11 weeks retain a certain level of H3K9me3, a constitutive heterochromatin marker, indicating its role in hPGC development.^171^ Histone lysine demethylase KDM2B would regulate the demethylation of histone marks, for example, H3K4me3 and H3K36me2 for hPGCLC specification.^224^ A recent report indicates that the hominidae-specific transposable elements (LTR5Hs) are expressed in both hPGCs and hPGCLCs, which are involved in chromatin accessibility and localized DNA demethylation. LTR5Hs retain an open chromatin state for binding by key PGC factors, including NANOG, TFAP2C, SOX17, and SOX15 after hPGCLC induction, and serve as TE embedded enhancers necessary for germ cell specification.^225^ In addition, LTR5Hs play an important role in the gene regulatory network shared between hPGCLCs and naïve ESCs.^226^ Thus, hPGC development is a complexly coordinated process involved in both genetic and epigenetic regulations, along with the spatiotemporal dynamic change of these regulators (Fig. 3).

### Migration and colonization of hPGCs

#### Migration route

PGCs are migratory cells during embryogenesis, which originate from the epiblast, move toward, and finally colonize the developing genital ridges. The PGCs eventually participate in gonad construction, together with somatic cells from intermediate mesoderm and visceral mesoderm.^39^ Our knowledge about mammalian PGC migration is mostly drawn from mPGCs in mice. After their specification in the epiblast, at around E8, the mPGCs begin to move actively into the visceral endoderm, go through the hindgut at E9.5, and during the E10.0–E10.5 period, migrate directionally from the dorsal body wall into the genital ridges.^15,227–231^ Wylie’s group clearly recorded the migration process by time-lapse analysis of living mPGCs from OCT4:GFP transgenic mice.^15,227^ In humans, the hPGC migration is mainly observed by morphology, histochemistry, and immunohistochemistry using the PGC markers, including alkaline phosphatase,^19^ glucosaminoglycans,^231^ and OCT4.^232^ The migration route from the hindgut epithelium towards the genital ridges is generally as follows, starting at around four weeks, out of the wall of the hindgut, through the dorsal mesentery to the midline of the dorsal wall, finally migrating into the developing gonads at 6 weeks^14,17,231–233^ (Fig. 2).

#### Signaling pathways and regulations for hPGC migration

Accumulating evidence shows that hPGC migration is both active and passive. Active movement of PGCs is considerable, although the migration along with passive translocation,^16,229^ as evidenced by (1) possessing pseudopodia,^17^ and (2) guiding of signaling molecules.^234^ For example, culture in vitro showed that the genital ridge tissue from 8.5 dpc mouse embryos could attract mPGCs and exert long-range effects on the migrating population of mPGCs.^235^ Screening for the factors involved in PGC migration has identified a number of signaling molecules essential for the migration from a variety of animals, including *Drosophila*,^138,236^ *Xenopus laevis*,^237^ zebrafish,^119,238–240^ chicken,^119,241^ and mice.^15,227,230,242–251^ Several signaling pathways of PGC migration are conserved in humans. Main signaling molecules and their pathways involved in PGC migration include SDF1–CXCR4, KIT-KITLG, HMGCR, and cholesterol. In addition, the extracellular matrix and sympathetic nerve fibers of the autonomous system play important roles in PGC migration.^231,232,251,252^

#### SDF1–CXCR4 signaling

SDF1 (also known as CXCL12) is a member of the alpha chemokine protein family. It is expressed in the body wall mesenchyme and genital ridges and acts as the ligand for the G-protein coupled receptor, and chemokine receptor 4 with the C-X-C motif (CXCR4) is expressed in migrating germ cells.^249^ Zebrafish have another SDF1/CXCL12 receptor, CXCR7, which is also crucial for PGC migration toward their targets.^253^ In mice, mPGCs have the cell-surface expression of the receptor CXCR4, and loss of the ligand SDF1 results in a delayed migration of mPGC.^244^ Embryos carrying targeted mutations in the receptor CXCR4 show defects in PGC migration and a reduced number in the genital ridges.^249^ Thus, the interaction through direct binding of SDF1 with CXCR4 plays a critical role in the directed migration of PGC towards the genital ridges. In fact, SDF1/CXCR4 pathway has also involved the migration of various cell types in humans, including cancer cells.^254^ TCam-2, a human germline seminoma, has a global similarity in gene expression pattern with hPGCs and hPGCLCs, including expression of chemokine members, CXCR4 and CXCR7, in addition to hPGC markers SOX17, BLIMP1, and CD38.^20,255^ CXCL12 supplement on matrigel-simulated basement membrane in culture shows a greater cell invasion in TCam-2.^256^ Nevertheless, direct evidence of SDF1–CXCR4 signaling in hPGC migration in vivo remains to be explored.

#### KIT-KITLG signaling

KIT is a receptor tyrosine kinase expressed on PGC surface for migration, and upon activation by its cytokine ligand KITLG in somatic cells, KIT phosphorylates intracellular proteins that could play a role in PGC migration. PGC motility and survival require both KIT and its ligand KITLG.^257^ Mice homozygous for KIT mutation are usually sterile, and their mPGCs are markedly reduced in number and showed a delayed and ectopic migration.^258^ The ligand steel (KITLG) is continuously expressed by somatic cells surrounding PGCs throughout migration,^234^ and the lacking KITLG results in cessation of motility and, finally, death of the ectopic germ cells, suggesting that KITLG protein promotes PGC migration.^242,257,259^ In addition, the transmembrane protein steel favors PGC adhesion to somatic cells via KITLG-KIT interaction, which may be independent of KITLG-induced tyrosine autophosphorylation of KIT receptor.^248^ Nevertheless, the culture of 11.5 dpc mPGCs showed that the ligand KITLG and 740Y-P peptide (an activator of PI3 kinase) rapidly increased autophosphorylation of its receptor KIT and caused phosphorylation of the serine–threonine kinase AKT through the action of PI3K and stimulated PGC migration, while the inhibitor of PI3K (LY294002) and inhibitor of the MEK/ERK signaling (U0126) impaired the PGC migration.^260^ In humans, genome-wide association studies have revealed that KITLG on chromosome 12 is a key susceptibility locus for testicular germ cell tumors in the populations of UK^261^ and US.^262^ An abnormally high expression of KIT has been observed in both testicular germ cell tumors and malignant ovarian.^263,264^ High expression of KIT is also detected in extragonadal testicular germ cell tumors, indicating an association of ectopic PGCs with extragonadal tumors, for example, in the central nervous system.^265^ It is worth mentioning that extragonadal tumors have been linked to the KIT-KITLG signaling, because of aberrantly migrated ectopic PGCs.^266^ These data indicate the importance of KIT-KITLG signaling in hPGC migration and neoplastic transformation of the germ cells derived.

#### HMGCR and cholesterol

Hydroxymethylglutaryl coenzyme A reductase, HMGCR (also known as HMGCoAR or LDLCQ3), is a rate-limiting enzyme for the cholesterol synthesis pathway. HMGCR inhibition by atorvastatin, an HMG-CoA reductase inhibitor that also has the ability to effectively decrease blood lipids, exhibits germ cell migration defects in zebrafish embryos, which can be rescued by mevalonate, the product of HMGCR activity.^239^ In mice, the genital ridges could accumulate high levels of cholesterol by localized uptake, and inhibition of the HMGCR activity in the culture of the genital ridges resulted in defects of germ cell survival and migration, suggesting that cholesterol is required for PGC survival and motility.^243^ Although direct evidence of the roles of cholesterol in hPGC migration is lacking in humans, recent studies show that cholesterol has essential roles in the specific ligand binding mode in the CX3CR1 chemokine.^267^ Given that the receptor CXCR4 for the ligand SDF1 shares a similar structure of the key region ECL2 with CX3CR1,^267^ thus, cholesterol molecules probably play essential roles in the receptor activation of SDF1–CXCR4 signaling for hPGC migration. As a novel link between cholesterol metabolism and hPGC development, this is a particularly interesting topic, which remains to be elucidated further.

#### hPGC-related diseases: infertility and cancer

**Fig. 4:**
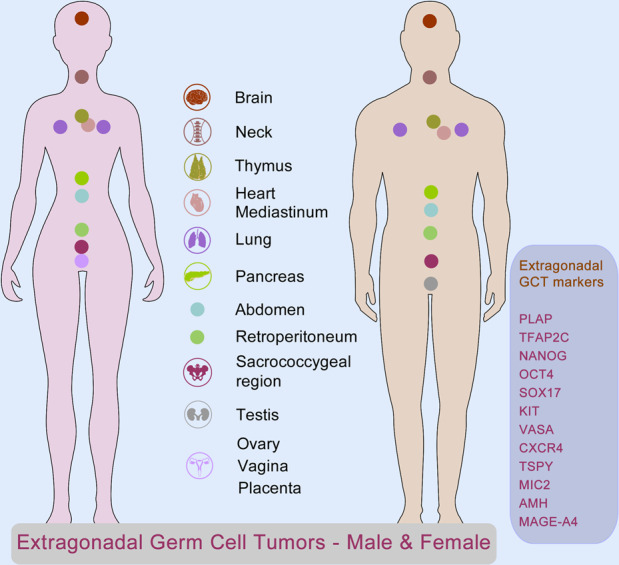
Extragonadal germ cell tumors (GCTs) in humans, related to PGC mismigration. As being pluripotential tumors, extragonadal germ cell tumors occur in a wide range of organs from central nervous system to ovary and testis indicated in the central panel, with expression of PGC markers, including PLAP, TFAP2C, NANOG, OCT4, SOX17, KIT, VASA, CXCR4, TSPY, MIC2, AMH, and MAGE-A4 listed in the right panel. Some elements of the figure were derived from Soehui

The hPGC development is an essential event during embryogenesis. Dysregulation of hPGCs in origin, migration, colonization, and differentiation will lead to major diseases in humans. The main types of diseases related hPGCs include infertility and cancer. Infertility is one of the main disorders in humans, which is mentioned throughout this topic. Thus, we will mainly discuss human germ cell tumors (GCTs) as follows. Ovarian cancer is one of the five deadliest cancers in women.^4^ Human GCTs are generally derived from germline cells, stem cells in particular, in the early embryos. GCTs occur not only in gonads (ovary and testis) but also in various organs in humans, although the most common types of GCTs are testicular germ cell cancer and ovarian cancer. Human germ cell tumors have been classed into seven GCT types, from type 0 to type VI, based on their developmental potential.^268^ Extragonadal GCTs are involved in a wide range of organs, for example, brain, head/neck, heart/mediastinum, lung, thymus, sacrococcygeal region, abdomen, retroperitoneum, vagina, and placenta, which are also sites of germ cell tumors^266,268,269^ (Fig. 4). It is widely accepted that extragonadal GCTs mainly originate from mismigration of hPGCs that failed to undergo apoptosis.^265,270–272^ Approximately 3% of malignant pediatric tumors are GCTs, and most of them are brain cancers.^265,273,274^ Interestingly, central nervous system GCTs express pluripotency marker genes PLAP, TFAP2C, NANOG, and KIT, in addition to markers for Sertoli/granulosa cells, MIC2 and AMH, and cancer-related markers MAGE-A4 and TSPY.^265^ Mutations are detected in intracranial germ cell tumors, including KIT, its downstream mediators KRAS and NRAS, copy number gains of the AKT1, and tumor suppressor BCORL1.^275^ In addition, mismigration of hPGCs in progenitor cells in the pancreas during early embryogenesis has been suggested as the main pathogeny of mucinous cystic neoplasms of the pancreas in humans.^276^ In general, as being pluripotential tumors, it is a very important and common understanding that the GCTs express germline markers, including OCT4, SOX17, NANOG, VASA, KIT, CXCR4, and TSPY, which may be used for diagnosis and treatment of extragonadal GCTs.^263,265,268,277–281^ For example, knockdown of CXCR4 expression suppresses proliferation, adhesion, and migration,^282^ and CXCR4 antagonists will be a promising therapy in antitumor activity in patients with various malignancies.^278^

In addition, hPGC-related disorders include other types of diseases. For example, Fanconi anemia, a recessive congenital disease, has characteristics of progressive bone marrow failure, mismigration of hPGCs, and predisposition to cancer, including acute myeloid leukemia and squamous cell carcinoma. FANC family of genes and related DNA interstrand crosslink pathway are identified for Fanconi anemia pathology,^283^ and FANCG is responsible for the PGC migration.^245^ Overexpression of hPGC marker gene PRDM14 is detected in lymphoblastic lymphomas,^284^ suggesting PRDM14 as a proto-oncogene involved in lymphoblastic lymphoma formation. It has been suggested that depletion of PRDM14 expression may be an effective and radical therapy for solid cancers.^285,286^

Most germ cell tumors are not caused by gene mutations, but instead by reprogramming their germ cells of the origin in the target niches. For example, testicular germ cell tumors prefer retention of PGC-lineage erasure of both maternal and paternal DNA imprints^272,287,288^ and histone modifications like H3K27ac.^289^ Somatic gene mutations and chromosomal mutations may also result in some germ cell tumors. For example, isochromosome 12p is common in seminomas and non-seminomas.^280,290–294^ KIT-KITLG mutations or their signaling activation,^262,264,275,281,295–299^ deletions of genes (e.g. SOX17, the gr/gr deletion on chromosome Y),^279,300^ structural variation, duplication, and loss of chromosomes,^301–304^ and aneuploidy^302^ are frequently detected in germ cell tumors. Recent reports show that super-enhancers are preferentially amplified in ovarian cancer.^305^ Indeed gene amplification occurs frequently in ovarian cancer.^306^ In addition, loss of the PGC gene TFAP2C leads to a high rate of germ cell tumors in mice, resembling pediatric Type I germ cell tumors in humans.^201^ DMRT1, a spermatogonia marker, is highly expressed in germ cell neoplasia in situ, and drives in vivo reprogramming and propagation of GCT-like tumor cells,^307^ indicating a shared feature of DMRT1-mediated reprogramming in germ cell tumors. Furthermore, germ cell tumors might be composed of somatic tumor cells and PGC-like tumor cells,^308^ which may bring difficulties to medical treatment. In clinics, cisplatin-based chemotherapy is a major means for the treatment of GCTs, which has a high cure rate.^268^ However, resistance to cisplatin often occurs in a proportion of 10–20%.^280^ The deubiquitinase USP11 is an important determinant of ovarian cancer chemoresistance.^306^ JMJD6 inhibitor SKLB325 has a significant effect in suppressing proliferation and promoting apoptosis of ovarian cancer cells.^309^ New targeted treatment approaches are needed for germ cell tumors. In particular, diagnosis and treatment based on pluripotency markers and targets of GCTs will be promising strategies. For example, the hPGC gene LIN28B plays a very important role in the inhibition of apoptosis through regulation of the AKT2/FOXO3A/BIM axis in ovarian cancer cells,^310^ indicating a novel target based on hPGC pluripotency in the diagnosis and therapeutics of ovarian cancer.

## Human PGCLCs, induced PGC-like cells

It is inaccessible to early hPGCs in vivo for the study of the human PGC development because of ethical issues. Fortunately, in vitro induction systems for differentiating hESCs/iPSCs into hPGC-Like Cells (hPGCLCs) have been established,^20–22^ which are not only a way of circumventing the issues, but also provide an approach to producing functional human gametes from hPGCLCs in vitro in the future. The in vitro reconstitution of human germ cell development will be instrumental in developing innovative medical applications in infertility and cancer.^311,312^ Thus, hPGCLC advances facilitate our understanding of human germ cell development and provide a new therapeutic means for treating infertility and cancer.

### Features of hPGCLCs

Early studies in mice showed that ES cells possess the ability to contribute to the germ cell lineage when cultured in vitro.^313–315^ Mouse ES cells derived from the ICM (inner cell mass) of the blastocyst are coaxed to differentiate into oogonia and sperm cells in vitro.^314,315^ Following the studies of the induced PGCs in mice, human ICM cells were also induced to differentiate into embryoid bodies, and some of the induced cells expressed markers of germ cells, including VASA,^316^ indicating that human ES cells could also be induced into the PGC-like cells in vitro. Based on the principle of hPGC development, robust approaches for hPGCLC specification in vitro from germ cell competent hESCs/hiPSCs under defined conditions have been developed.^20–22^ In summary, hPGCLCs possess several features of in vivo hPGCs. (1). These hPGCLCs show a similar pattern of gene expression to that of early hPGCs, including core PGC genes, SOX17, BLIMP1, and TFAP2C, but do not express late PGC markers including DAZL and DDX4.^20,21^ (2). Both hPGCLCs and hPGCs also share expression of pluripotency genes (NANOG and OCT4) and cell-surface markers (CD38, EPCAM, and ALPL).^20^ (3). The hPGCLCs exhibit upregulation of 5hmC (5-hydroxymethylacytosine) and TET1(a demethylase that belongs to the TETs), and a decline in the expression of de novo DNA methyltransferase 3 A and 3B (DNMT3A and DNMT3B),^20,171^ indicating an early pattern of DNA demethylation. Global loss of DNA methylation in the hPGCLC genome reveals the progress of epigenetic reprogramming similar to hPGCs in vivo.^22^ (4). Thusly, the hPGCLCs would correspond to early-stage hPGCs in vivo and probably represent pre-migratory hPGCs. (5). Finally, the hPGCLCs and hPGCs share a functional similarity in differentiating into oogonia and prospermatogonia.^317–319^ In induction culture systems, hPGCLCs are cultured with mouse fetal testicular somatic cells in long-term cultured xenogeneic reconstituted testes^317^ or with mouse ovarian somatic cells in xenogeneic reconstituted ovaries.^318,319^ hPGCLCs are also co-cultured with somatic cells from postnatal rat testes.^320^ These somatic cells provide an appropriate niche for hPGCLCs to mature into oogonia or prospermatogonia similar to those of hPGC differentiation in vivo, respectively. Nevertheless, differentiation in vitro from hPGCLCs to mature gametes (ova and spermatozoa) and function testing remain to be explored further.

### Methodologies for inducing hPGCLCs

hPGCLCs can be induced from both embryonic stem cells (hESCs) and induced pluripotent stem cells (hiPSCs). Two strategies for inducing hPGCLCs from hESCs and hiPSCs have been developed, the iMeLCs strategy and the 4i strategy.^20,21^ The basic principle of both strategies is based on the development rules of hPGCs, maintaining hPGC pluripotency, inducing hPGC fate, and inhibiting endodermal and other somatic genes.

#### The iMeLCs strategy

**Fig. 5:**
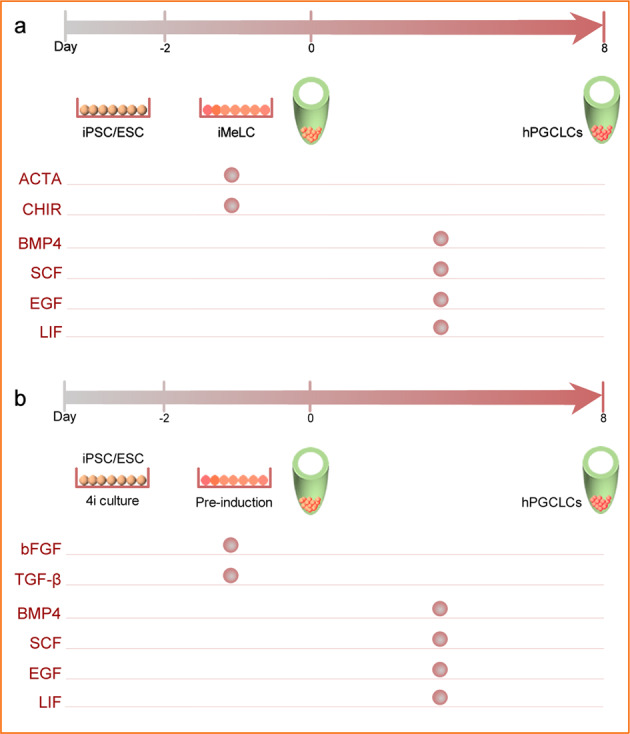
Methodology of hPGCLCs induction. hPGCLCs are induced from both embryonic stem cells (hESCs) and induced pluripotent stem cells (hiPSCs). **a** The iMeLCs strategy. **b** The 4i strategy. Induction culture timelines and added factors are indicated on the upper and the left in each panel, respectively

The strategy is a two-phase induction process from hiPSCs to iMeLCs and then to hPGCLCs (hiPSCs- iMeLCs- hPGCLCs)^21^ (Fig. 5a). During the first phase, hiPSCs are cultured under a conventional condition, and induced into incipient mesoderm-like cells (iMeLCs), a similar state to EpiLCs induced from ESCs/iPSCs in mice.^321^ EpiLCs bear a cellular state similar to pregastrulating epiblasts with high competence for the PGC fate.^321^ In the phase, Activin A and CHIR (a WNT signaling agonist^322^) are added to KSR medium to stimulate hiPSCs for 48 h, which is critical for hiPSCs to acquire a capacitated iMeLC state.^21^ iMeLCs are incipient mesoderm/primitive streak-like cells, express genes for pluripotency and mesoderm, but do not express PGC markers.^21^

During the second phase from iMeLCs to hPGCLCs, the iMeLCs are cultured in GMEM + 15% KSR with BMP4, SCF, LIF, EGF, and Y-27632. The Y-27632 is a selective inhibitor of p160-Rho-associated coiled-coil kinase (ROCK) and plays roles in antiapoptosis and increases cloning efficiency.^323^ For hPGCLC induction, BMP4 is essential, which acts through activin receptor-like kinase 2/3 and upregulates GATA3.^21,195^ SCF, LIF, and EGF play additive roles in the proliferation and survival of hPGCLCs.^21,321^ The hPGCLCs are induced by plating iMeLCs into a well of a low-cell binding U-bottom 96-well plate. The induced hPGCLCs exhibit upregulation of the regulators for hPGCLC specification, including TFAP2C, PRDM1, SOX17, SOX15, KLF4, KIT, TCL1A, and DND1, whereas downregulated genes are those involved in pattern specification processes and neuron development.^21^ Epigenetic change shows low levels of H3K9me2 and DNA methylation, including the imprint erasure of H19, but imprints of MEG3, KCNQ1, and PEG10 are not affected,^21^ which are similar to those of mPGCLCs.^198,321^ These hPGCLCs also express cell-surface markers EpCAM and INTEGRINα6,^21^ which can be used to identify and purify the hPGCLCs using immunofluorescence and fluorescence-activated cell sorting analyses.

#### The 4i strategy

To be a competent state for hPGC fate, hESCs and iPSCs are first cultured on MEFs with 4i (inhibitors for MAPK, GSK3, p38, and JNK) and preinduced by TGFβ and bFGF for 2 days.^20^ The inhibition culture has been used in hPSC culture, which represents a naive state of human pluripotency.^324,325^ These preinduced cells are further induced into hPGCLCs by adding BMP2/BMP4, LIF, SCF, EGF, and Y-27632.^20^ The cells are generally induced in ultra-low cell attachment U-bottom 96-well plates. In the induction system, 4i culture is a key step, which makes the cells to be a competent state for hPGC fate (Fig. 5b). The induced hPGCLCs express key hPGC genes, including SOX17, BLIMP1, TFAP2C, PRDM14, STELLA, TNAP, and KIT, pluripotency genes, OCT4 and NANOG, and cell-surface markers, TNAP and CD38.^20^ The proportion of hPGCLCs (both TNAP and CD38 positive cells) in the culture of day 4 embryoids induced from 4i hESCs is close to 46%,^20^ indicating a high competency for hPGCLC fate in the induction system. The combination of cell-surface markers TNAP and CD38 can also be used to identify and purify the hPGCLCs using immunofluorescence and fluorescence-activated cell sorting analyses.

### hPGCLCs and infertility treatment

**Fig. 6:**
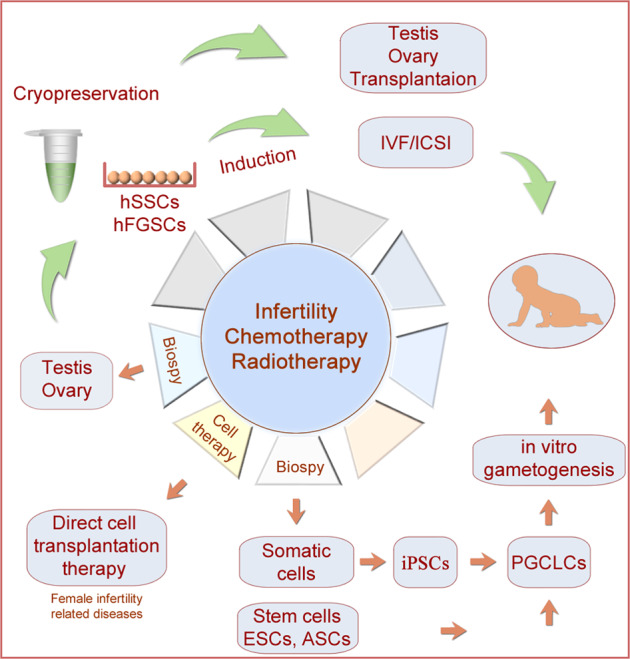
Fertility preservation and treatment of infertility and cancer in humans. Germ cell/gonad tissue transplantation is an alternative therapeutic means for treating infertility, and it is also a promising treatment strategy for both pubertal and prepubertal boys/girls diagnosed with cancers who will suffer from irradiation and chemotherapy. Cell sources for transplantation could include germ stem cells and hPGCLCs. As new progress in cell induction, transplanted cells might come from somatic cells of patients, which can be induced into gametes via hPGCLCs in the future. Some parts of the figure were drawn by Figdraw

Infertility affects over one-fifth of human couples worldwide.^10,11^ An increasing tendency to postpone child-bearing age often leads to difficulty to get children. There is a subsistent need for infertile patients who have an alternative choice, in vitro fertilization (IVF) with gametes derived from stem cells, or even somatic cells. There are great attempts to induce meiosis and haploid cells using hESCs/hiPSCs at different induction conditions.^326–330^ However, the induction is inefficient. hPGCLCs will be among the most promising cell types for producing gametes. hPGCLCs can be induced to differentiate into both oogonia and prospermatogonia by co-culturing with mouse embryonic ovarian or testicular somatic cells, respectively.^317–319,331,332^ Both oocytes and spermatozoa will be obtained via hPGCLCs induction for the treatment of infertile females and males. As mentioned above, hPGCLCs could also be induced from iPSC derived from somatic cells. Thus, in principle, oocytes and spermatozoa can be produced from somatic cells, not only from germline cells, in the future (Fig. 6). Of course, somatic cells as a new source of gametes via hPGCLCs will change our understanding of the continuity of life through germ cells,^23^ which needs wide discussions before application. Technology for the mouse in vitro gametogenesis has been closer to establishment. The oocytes induced from mPGCLCs are subjected to IVF, and viable pups have been obtained.^333–335^ The in vitro oogenesis system might also be used to explore molecular mechanisms for genetic diseases, for example, chromosomal aneuploidy.^336^ In addition, mPGCLCs start proper spermatogenesis after being transplanted into the testis, and relevant offspring are produced via IVF.^321^ Moreover, complete in vitro meiosis to generate male gametes from mouse ESC-derived mPGCLCs has also been reported.^337^ Spermatid-like cells derived from the mPGCLCs are subjected to intracytoplasmic injection into oocytes, and viable and fertile offspring have been obtained.^337^ The basic framework of human in vitro gametogenesis is roughly the same as that in mice, and further advances will benefit diagnosing, modeling, and treating infertility in humans.

## Adult germline stem cells

### Spermatogonial stem cells for spermatogenesis

#### Features of human SSCs

Both spermatogonial stem cells (SSCs) and female germline stem cells (FGSCs) are adult pluripotent stem cells (ASCs) in the germline. SSCs are pluripotent stem cells for generating spermatozoa in the testis, while FGSCs are newly identified pluripotent stem cells for producing oocytes in the ovary. Once gonadal colonization, hPGCs cease to proliferate, and the cells are called gonocytes or prespermatogonia in males.^338^ hPGCs will differentiate directly into fetal state 0-like spermatogonia starting at week 14 after fertilization.^339^ In humans, spermatogenesis begins 10–13 years after birth at puberty.^340^ During the first wave of spermatogenesis, some gonocytes resume proliferation, begin to move toward the periphery (basement membrane) of seminiferous tubules in the testis, and differentiate into spermatogonial stem cells.^341^ The SSCs are essential for generating spermatozoa throughout life. As germline stem cells, SSCs have features of stem cells, in addition to maintaining spermatogenesis. The main features of human SSCs include, (1) self-renewing to maintain SSCs population, (2) production of spermatogonia to support daily production of sperm cells,^342^ (3) SSC heterogeneity,^342–345^ as discussed below, (4) SSCs can transdifferentiate into other cell types, for example, oocytes, which is functional because the induced ovarian organoids derived from SSCs produced offspring.^346^ SSCs could be generated from induction from hPSCs,^347^ prepubertal SSCs are also induced to initiate meiosis and produce haploid germ cells in vitro.^348^ Somatic Sertoli cells are converted to become into SSCs by overexpression of DAZL, DAZ2, and BOULE,^349^ (5) SSCs are rare and account for about 0.02–0.03% of all cells in the testis,^350^ and (6) de novo mutations in SSCs increase as men age, which often are associated with congenital disorders.^351^

#### SSC heterogeneity and regulation

The earlier work has defined two types of A spermatogonia in humans, the A_dark_ and A_pale_ spermatogonia, which are considered undifferentiated stem cells, the reserve stem cells, and renewing stem cells, respectively.^352–354^ Single-cell RNA sequencing analysis of human testis has further exhibited spermatogonia heterogeneity by determining subtypes or states, and developmental trajectory.^343–345,355^ During the developmental process, SSCs first form progenitors that undergo proliferative expansion, then generate differentiated spermatogonia. Single-cell RNA clustering also shows three stages of spermatogonia, from SSCs to differentiating spermatogonia and then to differentiated spermatogonia.^345^ These spermatogonia at different developmental stages show three-dimensional chromatin architectural differences^356^ and express distinct marker genes.^343–345,355^ For example, SSCs cluster expresses markers, including GFRA1, RET, NANOS2, NANOS3, ZBTB16, SALL4, POU3F1, FGFR3, UTF1, PAX7, UCHL1, PLZF, and ID4, differentiating spermatogonia show expression of KIT, MKI67, DMRT1, and SOHLH1, indicating the proliferation of active spermatogonia, and STRA8, KIT, and MAGE-A4 are expressed in differentiated spermatogonia.^344,345,357–360^ The activation of EGR4, KLF6, KLF7, and/or SOX4 might be involved in the differentiation process from hPGCs to prespermatogonia.^175^ Nevertheless, functions in vivo of these SSC genes in humans remain to be determined further.

Using animal models, functions of important regulatory genes and pathways in human SSCs could be clarified. For example, in a mouse model, mTORC1 and FOXO1 signaling have been shown to be key regulators for regenerative undifferentiated spermatogonia.^361^ The hepatic stellate cell activation pathway is upregulated in SSCs, suggesting its role in the specification and maintenance of SSC fate.^343^ The SSC niche is another regulatory layer for SSC development. The seminiferous tubule and the interstitial tissue provide a local niche for SSCs.^362,363^ Somatic Sertoli cells are the supporting cells in the testis, and their differentiation is regulated by waves of transcription factors SRY, SOX9, AMH, and DHH.^364^ Sertoli cells finally generate and secrete specific factors for SSC development.^365^ The secreted glial cell-line-derived neurotrophic factor (GDNF) promotes the self-renewal of SSCs, while inhibiting their differentiation.^366^ GDNF activates RET tyrosine kinase in undifferentiated type A-spermatogonia collaborated with GFRA1, a ligand-specific co-receptor.^367,368^ Thus, GDNF signaling is essential for SSC self-renewal.^369^ CXCL12–CXCR4 signaling not only play an important role in PGC migration, as mentioned earlier but also in the establishment of the SSC niche. Sertoli cells express CXCR12, while SSCs express CXCR4 receptors on their membrane. Inhibition of CXCR4 signaling in mouse testes impaired SSC maintenance, leading to germline loss.^370^ Somatic Leydig cells are another type of supporting cells in the testis. Spatial transcriptome analysis shows distinct microenvironment compositions surrounding the undifferentiated versus differentiating spermatogonia between Leydig cells and Sertoli cells.^371^ Retinoic acid signaling regulates the differentiation process of the undifferentiated spermatogonia to differentiated spermatogonia.^372^ IGF1 and FGF9 signaling might also be associated with SSCs development.^373^ A recent report shows that H3K79 methyltransferase DOT1L is essential for SSC self-renewal in mice, which is associated with HOXC expression,^374^ indicating the importance of histone modifications in SSC regulation. At genomic DNA levels, ZBTB43 safeguards genomic integrity by regulating de novo DNA methylation at CG-containing purine–pyrimidine repeats, removing Z-DNA, and preventing DNA double-strand breaks in mouse prospermatogonia.^375^ It is interesting that ZBTB43 expression is also involved in cancer stemness in humans.^376^ In addition, male reproductive aging is associated with the capacity decline of SSC niche,^363,377^ thus, older males are often accompanied by a decrease in reproductive function.

#### SSC transplantation for fertility preservation in cancers and gene therapies

Spermatogonial stem cell transplantation is a promising approach to restore fertility for patients who need chemotherapy and radiation treatment, such as in cancer therapies, because these treatments often lead to damage to gonadal cells, thus infertility,^361,378,379^ although some antioxidants can reduce the damage to germ cells.^380^ Prepubertal boys who suffer from gonadotoxic treatment under pediatric cancer circumstances, e.g., acute lymphoblastic leukemia and testicular cancer, might cause sterile for the rest of their life.^381^ Autologous transplantation of SSCs or testicular tissue has been proposed as a strategy for fertility preservation and therapy^382–385^ (Fig. 6). In Europe, Canada, and USA, over 1033 young patients between 3 months and 18 years of age have already joined in fertility preservation by testicular tissue storage for late use.^382,386^ SSC transplantation has been considered in the fertility preservation of other genetic diseases, such as Klinefelter syndrome.^387^ Transgender women might also cryopreserve their germ cells before hormonal treatment, as a small percentage of transgender women have immature male germ cells.^388^

Testis-derived germ cell microinjection into seminiferous tubules of infertile recipients was first reported in mice, and transplanted cells colonized seminiferous tubules and initiated spermatogenesis.^389,390^ Germ cell transplantation in interspecies and intraspecies has been applied to zebrafish, rats, dogs, farm animals (goats, sheep, pigs, and cattle), nonhuman primates, and humans, in addition to mice.^391–408^ In primates, postpubertal SSC transplantation in infertile rhesus monkeys restored functional sperm production after puberty.^409^ In humans, the first clinical trial of testis-cell transplantation using cryopreserved single-cell suspension from patients’ testis with lymphoma before chemotherapy was reported in 1999.^410^ Nevertheless, it is difficult to evaluate the outcome, as endogenous spermatogenesis can occasionally escape from radiation or chemotherapy. Transplanted tissue/cells for fertility preservation may be testicular cell suspension, testicular prepubertal tissue fragments, or SSCs.^382^ SSCs can be isolated from the testis and proliferate in culture or induced from stem cells or Sertoli cells.^349,411–413^ Actual clinical implementation and safe should be carefully considered in the near future, for example, xenofree, clinical grade media, culture condition, and protocols.^414,415^ One of the concerned main issues about transplantation in cancer patients is the risk of reintroducing malignant cells present within tissue fragments to the patients. In fact, hPGCLCs will be a potential cell source for transplantation (Fig. 6). As mentioned earlier, hPGCLCs can be derived from iPSC, thus adult tissues from patients are an actual source of the cells, yet avoiding testis biopsy.

In addition, SSC transplantation is a promising treatment strategy for patients with genetic diseases, for example, Klinefelter syndrome, thalassemia, and drepanocytosis.^29^ For patients who carry gene mutations, their SSCs or hPGCLCs may be corrected before transplantation using CRISPR/CAS9 technology, which has potential applications for the treatment of genetic diseases in humans. Germline gene therapy via SSCs has achieved success in correcting an X-linked testis-expressed 11 (TEX11) mutation in mice with azoospermia phenotype.^416^ The mutant SSCs were isolated, and the TEX11 mutation was corrected by CRISPR-CAS9 technology. The final repaired SSCs were implanted back into the testis, which restored spermatogenesis in infertile males and gave rise to fertile offspring.^416,417^ The treatment technology might be used to cure azoospermia patients with TEX11 or other gene mutations in the future. However, the strategy needs to wait for a long-term discussion, public acceptance, and ethical argument, because of considerable ethical concerns for gene therapy through germline in humans.

### Female germline stem cells for oogenesis

In contrast to well-known SSCs, FGSCs are newly identified germline stem cells in mammals, including humans. It is reported that juvenile and adult mouse ovaries have mitotically active germ cells, but the same group subsequently indicates that both bone marrow and peripheral blood serve as a source of these germ cells in adulthood.^34,418^ FGSCs are identified in neonatal and adult ovaries, which are isolated from ovaries and differentiate into functional oocytes after transplantation into mouse ovaries.^25^ Thus, FGSCs are able to undergo postnatal neo-oogenesis,^25,35^ possibly providing oocytes for reproductive life. The finding of FGSCs has updated the traditional idea that the ovary possesses a finite oocyte reserve before birth in female mammals.^31–33^ Thus, proliferation and differentiation of FGSCs may replenish the gradual exhaustion of reserved primordial follicles throughout the female’s fertile life. It has been shown that infertility and death of women are partly attributed to ovarian function-related diseases, for example, polycystic ovarian syndrome, premature ovarian failure, and ovarian cancer.^419^ Applications of FGSCs in reproductive medicine have significant clinical implications in the treatment of female reproductive aging and ovarian function-related disorders. For example, FGSCs transplantation might be applied to patients with ovarian cancers or premature ovarian failure as a strategy for fertility preservation and therapy in females (Fig. 6). Actually, ovary tissue possesses several types of somatic stem cells yet, for example, human OSE (ovarian surface epithelium) stem cells, which express SOX-2 and SSEA-4, but FGSCs do not, although they can form oocyte-like cells in culture,^420^ and human VSELs, a very small embryonic-like stem cells with nuclear OCT4 expression and LGR5+,^421,422^ in addition to Thecal stem cells and granulosa stem cells.^423^ However, FGSCs are germline stem cells, which will be discussed as follows.

#### Features of FGSCs

As germline stem cells, FGSCs have characteristics of adult pluripotent stem cells, in addition to maintaining oogenesis. The main features of FGSCs in mice and humans are summarized as follows. (1) FGSCs show similar morphology to those of SSCs with large nuclei and little cytoplasm.^424^ (2) FGSCs isolated from neonatal mice display the string-forming cell configuration, and E-cadherin mediates the cell–cell contact at membrane connection sites.^425^ (3) FGSCs express membrane marker Fragilis/IFITM3, while MVH and alkaline phosphatase are mainly localized to the cytoplasm and also expressed on the membrane.^424,426^ (4) IFITM3, DAZL, MVH, OCT4, PRDM1/BLIMP1, and DPPA3 are markers for FGSCs, in addition to TERT and alkaline phosphatase, and cell cycle-related transcription factors c-MYC and EGR-1 are also expressed in FGSCs.^25,424,427^ However, NANOG, SSEA-1, and SOX2 are not expressed in both neonatal and adult FGSCs.^424^ In addition, OCT4, BLIMP1, and DAZL are common markers in both PGC and FGSCs. (5) FGSCs have high telomerase activity.^25^ (6) FGSCs could be converted to female ES-like cells under ESC culture conditions with the addition of vitamin C and valproic acid, which show similar characteristics to ESCs in genomic imprinting, formation of the three germ layers and chimeras, and germline transmission capacity.^428^ (7) FGSCs self-renew to maintain proliferation with vigorous mitosis capacity.^25,425^ (8) FGSCs have the capacity to produce normal oocytes to support the generation of fertile offspring after transplantation into mouse ovaries.^25^ Primordial follicle-like structures form in vitro co-culture with granulosa cells from neonatal mouse ovaries.^429^ Oocytes are also generated in xenografted human ovary tissue after being injected into adult human ovarian cortical tissue biopsies and then xenografted into NOD-SCID female mice.^427^ (9) Given the germline transmission ability of FGSCs, genome editing of FGSCs might be used to treat genetic diseases in humans and to alter specific traits in animals. For example, transgenic animals, through introducing genes with functional importance and commercial value into FGSCs have been obtained.^430–433^ Transgenic rats with *fat-1* gene, a *Caenorhabditis elegans* gene for the synthesis of N-3 polyunsaturated fatty acids from N-6 fatty acids, have been generated using FGSCs.^430^ N-3 polyunsaturated fatty acids are essential for human development, and their deficiency is associated with human diseases, including cardiovascular disease, hyperinsulinemia, and type 2 diabetes.^434–436^ Most mammals do not have *fat-1* gene in their genomes, thus cannot convert n-6 into n-3 polyunsaturated fatty acids, which should be acquired by food intake. The *fat-1* transgenic farm animals will provide an alternative food source of N-3 polyunsaturated fatty acids for humans.

#### Isolation and characterization of FGSCs

FGSCs exist in neonatal and adult mouse ovaries, especially enriched in neonatal ovaries of 1–3-day postpartum. To isolate the FGSCs, the two-step enzymatic digestion method (collagenase and trypsin) is often used for the efficient digestion of ovary tissue. MVH- or Fragilis-positive cells are then separated by MACS or FACS technology using antibodies against MVH^25,427^ or Fragilis,^426^ respectively. The germline-specific Fragilis is a membrane marker in FGSCs, thus, the efficiency of FGSC purification using anti-Fragilis and FACS technology is higher than that using anti-MVH.^426^ Differential adherence selection with passaging enrichment from postnatal ovaries without any antibody has also been used to isolate FGSCs.^425^ FGSCs are often cultured in MEM‑α medium supplied with FBS, non-essential amino acids, transferrin, insulin, EGF, GDNF, and bFGF on an inactive STO feeder layer.^25^ Isolated FGSCs are characterized by testing FGSCs markers, OCT4, MVH, IFITM3, DAZL, and BLIMP1, and differentiating into oocytes in vivo or in vitro. As stem cells, the proliferation capacity of FGSCs should be tested using mitotic markers, including cell cycle-related transcription factors c-MYC and EGR-1. Due to a string-forming characteristic, the mitotic ability may be tested using the mitotic antagonistic agent mitomycin C to treat the cells, which results in a decrease in string-formation.^425^ Germline transmission and oogenesis capacity are essential functional tests, including the ability to produce fully functional oocytes and fertile offspring after transplantation into chemotherapy-damaged mouse ovaries.^25^

#### Regulation of FGSCs development

FGSCs not only self-renew but also differentiate to initiate meiosis. During FGSC development, they first differentiate into germinal vesicle oocytes, then into the prophase of meiosis II via the prophase of meiosis I processes. In these developmental processes, the FGSC genome undergoes dramatically reorganization.^437^ In addition, the X chromosome shows a smaller proportion of the active compartment in comparison with that of autosomes, because the X inactivation might take place.^437^

The stem cell niche in the ovary is an important aspect of the regulation of FGSCs development, which provides essential microenvironments and signaling pathways for FGSCs. Disruption of the niche or related signaling leads to stem cell loss.^438,439^ The niche aging in the ovary is associated with the decline in ovarian reproductive function.^440–442^ CADHERIN-22, a member of the cadherin superfamily, promotes FGSC self-renewal through interaction with the JAK and β-CATENIN.^443^ Meanwhile, CADHERIN-22 enhances PI3K-AKT3 signaling, thus, upregulating the expression of N-MYC and CYCLIN, and GDNF-GFRA1 activates AKT3 via PI3K or SFK, subsequently promoting self-renewal of FGSCs.^444^ GSK3 inhibitor BIO promotes proliferation of FGSCs through activation of β-CATENIN and E-CADHERIN,^445^ indicating an important role of GSK3 signaling in FGSCs proliferation. The Hippo effector YAP1 also regulates the proliferation of FGSCs in mice.^446^ In addition, Hedgehog signaling pathway plays an essential role in FGSCs development. Inhibition of the hedgehog signaling pathway with GANT61 leads to follicular atresia and reduction in FGSC proliferation capacity in the mouse ovary and in vitro culture of FGSCs.^447^ Accumulated evidence shows the importance of the hedgehog signaling in ovary development,^448–455^ supporting the role of the hedgehog signaling in FGSCs development.

Anti-cancer agent C89, one kind of benzoborazoles, induces FGSC autophagy by inhibiting the activity of Akt and PI3K in vitro, thus inhibiting the proliferation of FGSCs.^456^ ZCL-082, another kind of benzoborazoles, has a similar effect in promoting autophagy and inhibiting the proliferation of FGSCs, but via regulating GAS5(long noncoding RNA)/miR-21a expression.^457^ Whereas, spermidine induces cytoprotective autophagy via inhibition of AKT/mTOR phosphorylation in FGSCs.^458^ The AKT signaling pathway is activated by daidzein through upregulating the stem cell growth factor CLEC11A, thus promoting FGSC proliferation.^459^ It has been shown that autophagy regulation is closely associated with testis development, spermatogenesis,^460^ ovary development,^461–464^ oogenesis, and gonad diseases.^465–469^ Thus, regulation of the autophagy pathway in FGSCs through small molecules will be promising and potential therapeutic targets for ovary diseases, for example, premature ovarian failure and ovarian cancers.

#### FGSCs implantation and fertility preservation

As germline stem cells for females, FGSCs provide a new strategy for preserving fertility and delaying menopause, which will, of course, benefit female patients with reproduction dysfunctions (Fig. 6). FGSCs can be implanted into the ovary and initiate oogenesis in vivo for production of fertile offspring.^25^ FGSCs can also develop in vitro and differentiate into oocytes after injection into human ovarian cortical tissues xenografted into adult immunodeficient female mice.^470^ Alternatively, FGSCs are cultured into 3D ovarian organoids to produce oocytes for transplantation.^471^ in addition, functional oocytes are obtained through transdifferentiated from SSCs in vitro and also produce offspring in mice.^346^

In the clinic, cryopreserved ovarian tissues for females are being carried out.^472^ Autografting of frozen-thawed ovarian tissue fragments is already used to restore fertility from both adult^473^ and prepubertal, a 9-year-old girl suffering from thalassemia and a girl with sickle-cell anemia at age 14 years.^474,475^ A clinic study shows a high live birth rate (33%) after transplantation of ovarian tissue fragments.^473^ In humans, FGSCs have been obtained from scarce ovarian cortical tissues from follicular aspirates. These FGSCs differentiated into germinal vesicle stage oocytes in vitro for transplantation.^470^ Thus, the technology from scarce ovarian tissues to oocytes has clinical implications for fertility preservation for women of reproductive age before cancer treatment. In addition, FGSCs are transplanted into the ovary of infertile chemotherapy-treated mice, a premature ovarian failure model, finally restoring ovary function and generating offspring.^476^ This study provides a technology blueprint for clinic application in humans in the future, for example, in the treatment of premature ovarian failure, early menopause, and infertility, in addition to cancers.

## Conclusions and perspectives

We have displayed the fantastic features of germline stem cells in humans, self-renewing, generating ova or sperm cells via halving the genome, and passing genetic information from one generation to the next, in contrast to those of somatic cells. Germline stem cells are pluripotent from embryonic PGCs to adult germ stem cells, SSCs, and FGSCs, with different developmental states. Later, they are the only cells to undergo meiosis. hPGCs are specified in the early stage of embryos, then migrate into the genital ridge, where they meet somatic gonadal cells (e.g., Sertoli cells and Leydig cells in XY embryos, granulosa cells and theca cells in XX embryos) together to assemble testis or ovary, respectively. The XY embryos of age between 41 and 44 days start to express the sex-determining gene SRY on Y chromosome,^477^ which triggers differentiation of bipotent gonad into the testis, otherwise, in the XX embryos without SRY, the gonad will differentiate into ovary.^478^ In XY embryos, SSCs gradually develop and start the first time of meiosis for spermatogenesis in adolescent boys at the age of 10–13 years,^340^ while XX embryos initiate meiosis for oocyte production. FGSCs are newly identified germline stem cells in neonatal and adult ovaries, which support self-renewing and differentiating into oocytes for the production of offspring.^25^ The finding of FGSCs demonstrates a new concept that adult women can continuously generate oocytes in their reproductive life. Notably, another important advance is that hPGCLCs can be induced in vitro from germ cell competent hESCs/hiPSCs under defined conditions.^20–22^ These advances have opened up new avenues to understand human germ cell development and provide new approaches to in vitro gametogenesis^23^ and therapeutic means for treating infertility and cancer (Fig. 6).

However, there are also multiple issues that need to be solved to understand human germline stem cell fate and develop new diagnosis and therapy approaches for medical applications. First, developmental mechanisms of hPGCs in vivo at the peri-implantation stage are not well known, because of the difficulty of access to human early embryos. This issue might partially be resolved through hPGCLCs developmental processes, adopting 3D organoid culture together with machine learning and artificial intelligence in particular, which will provide tractable in vitro models of human physiology and pathology.^471,479–481^ There is a general consensus that differences are remarkable in mechanisms underlying PGC development between mice and humans,^24,86,157^ while germ cell development in nonhuman primates better mimics the relevant processes in humans.^89–91,95,160,187,482^ Further studies using nonhuman primate models will provide new insight into germ cell development and differentiation. Second, cell therapy based on germline stem cells for infertility needs to be further explored, including selection and induction of donor cell type/state, cell transplantation, and quality control. Human in vitro gametogenesis has not been reached yet, although complete in vitro meiosis from mESC-derived mPGCLCs has been reported in mice.^337^ Nevertheless, hPGCLCs are a promising source for gamete production in vitro in the future, and more importantly, they can be induced from somatic cells.^23^ Nevertheless, social and ethical issues concerning in vitro gametogenesis and following IVF using these germ cells should be seriously discussed before applications in the clinic. Third, fertility preservation has also become a pressing issue.^483–485^ Several factors, including gonadotoxic therapies, environmental exposures, aging, genetic diseases, and cancers, might cause subfertility or infertility.^6,486,487^ Germ cell transplantation is a promising strategy for both pubertal and prepubertal boys/girls diagnosed with cancers who will suffer from irradiation and chemotherapy,^488–499^ because these therapies often lead to damage to SSCs and FGSCs of the patients.^483,500–510^ It is also important to consider the purity of transplanted germ cells, and cancer cell contamination must be completely eliminated.^511,512^ Of course, fertility preservation raises several ethical issues,^513–516^ which should be carefully considered with discussion and debate. Fourth, understanding extragonadal germ cell tumors has guided us to consider the diagnosis and treatments of cancers, extragonadal cancers in particular. Most extragonadal germ cell tumors occur in many organs other than the testis and ovary, for example, brain cancers.^517–523^ These cancers have some features of hPGCs,^524,525^ thus hPGC markers should be used to diagnose and treat these cancers in the future. Actually, targeting the WNT signaling pathway for cancer therapy has been in preclinical testing and clinical trials.^526–528^ Lastly, genome editing in germline stem cells in humans is a very cautious approach because of its germline transmission to the next generation. Genome editing has been used to correct gene mutations in mice and mimic human genetic diseases,^529–533^ and correct pathogenic gene mutations in human embryos.^534,535^ CRISPR-edited T cells in patients with cancers have been tested in clinical trials.^536–538^ Genome editing technology in the targeted therapy has shown a promising prospect for human diseases,^539,540^ especially, in fertility restoration in cancer survivors and prevention of paternal transmission of diseases. Yet, there are several major obstacles to be overcome, including off-targets, social, and ethical issues. Technically, precise gene editing, for example, spatiotemporal control of CRISPR/Cas9 editing^541^ and non-viral strategy,^542^ will provide new hope in medical applications in the future.
