# Correction To: Germline stem cells in human

**DOI:** 10.1038/s41392-022-01258-7

**Published:** 2022-12-03

**Authors:** Hanhua Cheng, Dantong Shang, Rongjia Zhou

**Affiliations:** Hubei Key Laboratory of Cell Homeostasis, College of Life Sciences, Renmin Hospital of Wuhan University, Wuhan University, 430072 Wuhan, China

**Keywords:** Cell biology, Molecular medicine, Pluripotent stem cells

Correction to: *Signal Transduction and Targeted Therapy* 10.1038/s41392-022-01197-3, published online 02 October 2022

In this article^1^, the legend in Fig. 3 has a typo, “Extraembryonic” inadvertently supplied and published as Extraembroynic; the figure should have appeared as shown below.

Also, there was a typo in the first paragraph in The iMeLCs strategy section, “GK15” replaced with “KSR”.

The original article has been corrected.
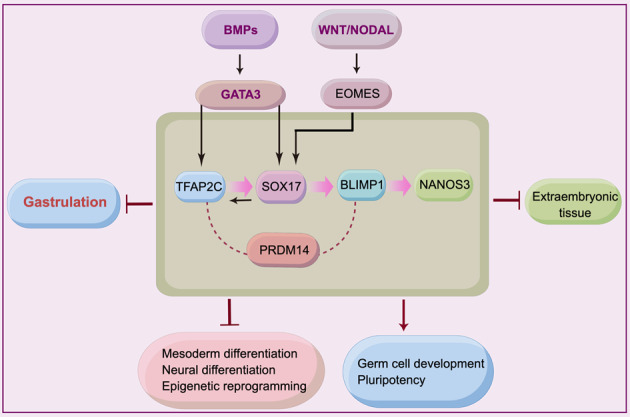


## References

[1] Cheng H, Shang D, Zhou R (2022). Germline stem cells in human. Sig. Transduct. Target. Ther..

